# Preoperative Carbohydrate Load Does Not Alter Glycemic Variability in Diabetic and Non-Diabetic Patients Undergoing Major Gynecological Surgery: A Retrospective Study

**DOI:** 10.3390/jcm13164704

**Published:** 2024-08-10

**Authors:** Robert Canelli, Joseph Louca, Mauricio Gonzalez, Michelle Sia, Maxwell B. Baker, Shama Varghese, Erin Dienes, Federico Bilotta

**Affiliations:** 1 Department of Anesthesiology, Boston University Chobanian & Avedisian School of Medicine, Boston, MA 02118, USA; robert.canelli@bmc.org (R.C.); varghese.shama@gmail.com (S.V.);; 2 Department of Obstetrics and Gynecology, Boston University Chobanian & Avedisian School of Medicine, Boston, MA 02118, USA; michelle.sia@bmc.org; 3 University of Vermont Larner College of Medicine, Burlington, VT 05405, USA; 4 University of New England College of Osteopathic Medicine, Biddeford, ME 04005, USA; 5 Department of Anaesthesiology, Critical Care and Pain Medicine, Policlinico Umberto I Teaching Hospital, Sapienza University of Rome, 00185 Rome, Italy; bilotta@tiscali.it

**Keywords:** preoperative carbohydrate loading, glycemic variability, enhanced recovery after surgery (ERAS), glycemic lability index, perioperative care, preoperative nutrition

## Abstract

**Background/Objectives**: Elevated glycemic variability (GV) has been associated with postoperative morbidity. Traditional preoperative fasting guidelines may contribute to high GV by driving the body into catabolism. Enhanced recovery after surgery (ERAS) protocols that include a preoperative carbohydrate load (PCL) reduce hospital length of stay and healthcare costs; however, it remains unclear whether PCL improves GV in surgical patients. The aim of this retrospective study was to determine the effect of a PCL on postoperative GV in diabetic and non-diabetic patients having gynecological surgery. **Methods**: Retrospective data were collected on patients who had gynecological surgery before and after the rollout of an institutional ERAS protocol that included PCL ingestion. The intervention group included patients who underwent surgery in 2019 and were enrolled in the ERAS protocol and, therefore, received a PCL. The control group included patients who underwent surgery in 2016 and, thus, were not enrolled in the protocol. The primary endpoint was GV, calculated by the coefficient of variance (CV) and glycemic lability index (GLI). **Results**: A total of 63 patients in the intervention group and 45 in the control were analyzed. GV was not statistically significant between the groups for CV (19.3% vs. 18.6%, *p* = 0.65) or GLI (0.58 vs. 0.54, *p* = 0.86). Postoperative pain scores (4.5 vs. 5.2 *p* = 0.23) and incentive spirometry measurements (1262 vs. 1245 *p* = 0.87) were not significantly different. A subgroup analysis of patients with and without type 2 diabetes mellitus revealed no significant differences in GV for any of the subgroups. **Conclusions**: This retrospective review highlights the need for additional GV research, including consensus agreement on a gold standard GV measurement. Large-scale prospective studies are needed to test the effectiveness of the PCL in reducing GV.

## 1. Introduction

Glycemic variability (GV) is the value used to track fluctuations in blood glucose concentration (BGC), notable for a greater correlation to endothelial dysfunction and oxidative stress than chronically elevated blood glucose levels [1]. High GV has also been associated with an increase in morbidity and mortality in hospitalized patients [2,3]. In the surgical population, elevated GV has been associated with postoperative morbidity, increased hospital length of stay, and increased hospital readmission rates [4,5,6].

Traditional preoperative fasting guidelines designed to prevent aspiration of gastric contents on induction of anesthesia may contribute to elevated GV by driving the body into a catabolic state and increasing perioperative insulin resistance. Moreover, the body’s stress response to surgery enhances gluconeogenesis and hinders glucose uptake, further exacerbating perioperative GV via the release of stress hormones and immune response suppression [7].

It has been postulated that timed ingestion of a preoperative carbohydrate load (PCL) prior to surgery, rather than traditional nil per os (NPO) guidelines, can improve postoperative GV by increasing endogenous insulin production and reducing catabolism. Moreover, PCL has been shown to improve postoperative muscle strength [8,9] and reduce postoperative pain scores [10,11]. Prescribing a PCL to patients up to 2 hours before surgery has become an integral part of institutional enhanced recovery after surgery (ERAS) protocols that bundle several interventions aimed at improving surgical outcomes [12,13,14]. Additionally, PCL has been included in consensus statements and practice guidelines from prominent international societies [15,16,17].

As has been shown for other types of surgery, ERAS protocols for gynecological surgery patients shorten hospital length of stay and reduce postoperative opioid consumption [18,19]; however, it remains unclear whether PCL administration works as intended to reduce GV in this surgical population. The aim of this retrospective study was to determine the effect of a PCL, as part of an institutional ERAS protocol, on postoperative GV in both diabetic and non-diabetic patients having major gynecological surgery. Additional postoperative outcomes influenced by PCL were also investigated.

## 2. Materials and Methods

### 2.1. Study Population

An institution-wide ERAS protocol was rolled out to all patients undergoing major gynecological surgery in February 2017. The ERAS protocol included ingestion of a PCL, which was documented on the preoperative nursing flowsheet. The PCL consisted of 56 g of carbohydrate in 32 ounces of liquid (Gatorade^®^, 400 kcal). Patients were instructed to drink 16 ounces of the PCL on the night before surgery and 16 ounces of the PCL up to 2 h prior to the surgical start time. Patients with type II DM were included in the ERAS protocol. The elements of the ERAS protocol can be found in Appendix A.

The Institutional Review Board approved this retrospective study on 21 February 2023. A request was placed to the clinical data warehouse (CDW) to obtain retrospective data on all patients who had major gynecological surgery within two periods corresponding to the timing of the institutional ERAS protocol rollout. The intervention group included those patients who underwent major gynecological surgery between 1 January 2019 and 31 December 2019 and were thus enrolled in the institutional ERAS protocol. The control group included those patients who underwent major gynecological surgery between 1 January 2016 and 31 December 2016 and, thus, were not enrolled in the institutional ERAS protocol. Patients included in the data set had at least one Current Procedural Terminology code that corresponded to hysterectomy. The CDW request included data that can be found in Appendix B. Patients who had at least two BGC measurements from the immediate postoperative period to postoperative day 3 were included in the final analysis.

### 2.2. Outcome Measures

Clinical characteristics were recorded, including age, race, comorbid conditions, including hypertension and diabetes mellitus, body mass index, and American Society of Anesthesiologists (ASA) physical status classification. The type of anesthetic used for maintenance of general anesthesia was recorded and included volatile anesthetic alone, volatile anesthetic plus propofol infusion, or total intravenous anesthesia with propofol plus remifentanil infusions.

The primary endpoint for this study was GV, measured in two different ways: coefficient of variance (CV) and glycemic lability index (GLI). The CV is equal to the standard deviation of postoperative BGC measurements divided by the mean BGC and expressed as a percentage. The GLI is measured by the total squared change in BGC per second, i.e.,

$$\sum_{n=1}^{N}\frac{{\left(BGC_{n\;}-\;BGC_{n\;+\;1}\right)}^{2}}{\left(h_{n\;+\;1}\;-\;h_{n}\right)}$$

where $BGC_{n}$ is the $n^{th}$ blood glucose concentration measured on the patient at time $h_{n}$, and n is the total number of BGC measurements from the patient within 3 days post-surgery.

Secondary outcomes included postoperative pain scores charted on the nursing flow sheet in the postanesthesia care unit (PACU) using the universal pain assessment tool and postoperative incentive spirometry measurements recorded in the PACU. Postoperative pain has been shown to be lessened after PCL ingestion [10,11]. Additionally, postoperative pain assessment in the PACU is routinely assessed and recorded in the medical record and available for retrospective analysis. With regards to incentive spirometry measurements, it should be noted that PCL has been shown to improve hand grip strength and pulmonary function as measured by peak expiratory flow rates postoperatively [8,9]. Unfortunately, neither of these measurements are routinely performed at this institution. However, incentive spirometry is routinely performed postoperatively to reduce postoperative pulmonary complications. Incentive spirometry measurements are documented in the medical record. Incentive spirometry has not been studied with respect to PCL ingestion; however, its relation to both muscle strength and pulmonary function lends itself to inclusion as a secondary outcome for this study.

A subgroup analysis was performed based on diabetes mellitus (DM) status to determine whether significant differences in GV between the control and intervention groups were present. Patients with DM in the control group were compared with patients with DM in the intervention group with respect to mean BGC, CV, and GLI. Similarly, non-diabetic patients in the control group were compared with non-diabetic patients in the intervention group with respect to the same variables.

### 2.3. Statistical Analysis

The data collected from this retrospective study were analyzed using a variety of statistical methods to determine whether significant differences exist in the primary and secondary outcomes between the control group and the intervention group. Descriptive statistics were utilized to summarize the study population’s characteristics, including means, standard deviations, and frequencies where appropriate. Inferential statistics, including two sample *t*-tests and chi-square tests or Fisher’s exact tests, when appropriate, were employed to assess differences between the two groups. All statistical analyses were conducted using the R software package, with a significance level set at *p* < 0.05 [20].

To determine the sample size required to detect a difference of 10% in average GV between the control and intervention groups, the following sample size calculation was used

$$n\;=\;{\left(Z_{\frac{\alpha }{2}}+Z_{\beta }\right)}^{2}\cdot \frac{\left(2\sigma^{2}\right)}{d^{2}}$$

where

- α = probability of a type one error, chosen to be 0.05;
- β = the power of the test, chosen to be 0.80 or 0.90;
- $\sigma^{2}$ = the variance of the populations, estimated here by the pooled variance with data from the retrospective study;
  ○ $s_{p}^{2}\;=\;\sqrt{\frac{\left(\left(n_{1\;}-1\right)s_{1}^{2}\;+\;\left(n_{2}\;-\;1\right)s_{2}^{2}\right)}{n_{1}+n_{2}-2}}=\;80.02$
- d = the desired effect size, in this case 10%.

Under this setting, a sample size of 126 (168) in both the control group and intervention group would detect a 10% difference with 80% (90%) power. Each subject would need at least 2 BGC measurements.

## 3. Results

### 3.1. Baseline Characteristics

**Figure 1:**
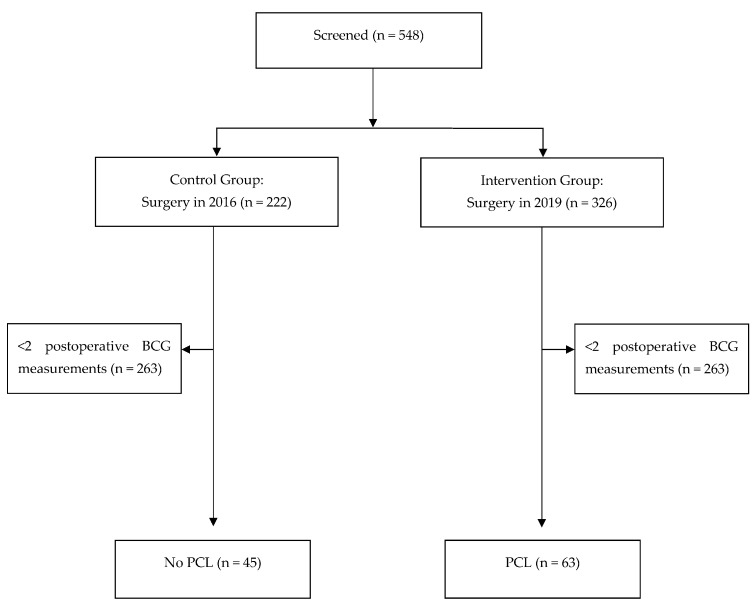
Flow chart showing the study inclusion criteria. BGC = blood glucose concentration, PCL = preoperative carbohydrate load.

A total of 548 patients met the screening criteria for having major gynecological surgery within the two stated periods: 326 in the intervention group and 222 in the control group. Of the 326 patients in the intervention group, 263 were excluded for having fewer than two postoperative BGC measurements. Similarly, of the 222 patients in the control group, 177 were excluded for having fewer than two BGC measurements. The final cohort of patients analyzed included 63 patients in the intervention group and 45 in the control group (Figure 1). The two groups were not matched, although the baseline characteristics of the two groups were well-balanced (Table 1). There were no significant differences in race listed, hypertension status, type 2 DM status, body mass index, or American Society of Anesthesiologists (ASA) physical status classification between the intervention and the control groups.

### 3.2. Anesthetic Type

There was a significant change in anesthetic management after the institutional ERAS protocol was introduced. The percentage of patients who received sevoflurane only for maintenance of general anesthesia reduced from 80% in the control group to 31.7% in the intervention group. Conversely, the number of patients who received an anesthetic technique that included sevoflurane plus propofol infusion increased from 15.6% in the control group to 60.3% in the intervention group (*p* = < 0.001) (Table 1).

### 3.3. Glucose Homeostasis

The mean BGC for patients in the intervention group was 152.6 ± 37.0 mg/dL, and for the control group was 158.4 ± 44.6 mg/dL (*p* = 0.48); the standard deviations were 29.3 and 29.6, respectively (*p* = 0.91). The GV was calculated using two methods: CV and GLI (Table 2). The CV for the intervention group was 19.3% ± 9.5 and for the control group was 18.6% ± 8.1 (*p* = 0.65). The mean GLI was 0.58 ± 1.6 for the intervention group and 0.54 ± 0.8 for the control group (*p* = 0.86). Neither the CV nor the GLI were significantly improved after ingestion of the PCL.

### 3.4. Secondary Outcomes

Postoperative pain was evaluated in the PACU using the universal pain assessment tool. Pain scores were not significantly reduced in the intervention group when compared with the control group (4.5 ± 3.4 vs. 5.2 ± 2.5, *p* = 0.23), although a trend toward significance was noted. Incentive spirometry was measured in the PACU postoperatively. The performance of patients using the incentive spirometer was not significantly better in the intervention group when compared with the control group (1262 ± 517 mL vs. 1245 ± 490 mL, *p* = 0.87) (Table 2).

### 3.5. Subgroup Analysis

A subgroup analysis of patients with and without type 2 DM was performed. The CV and GLI for patients in the control group without DM were 20.0% and 0.33, respectively. The CV and GLI for patients in the intervention group without DM were 19.4% and 0.15, respectively. The CV and GLI for patients in the control group with DM were 17.8% and 0.65. The CV and GLI for patients in the intervention group with DM were 19.3% and 1.08. There were no significant differences in GV for any of the subgroups (Table 3).

## 4. Discussion

This retrospective study revealed that PCL, as part of an institutional ERAS protocol, was not associated with a reduction in GV in patients undergoing major gynecological surgery. The lack of a significant difference between the intervention and control groups for the primary outcome was further supported by the secondary outcome results and the subgroup analysis of patients with and without DM. It was hypothesized that glucose homeostasis, in this case measured by CV and GLI, would improve with the addition of a PCL; however, that was not seen in this retrospective study.

Evaluating one specific element of a bundled protocol on patient outcomes is challenging and may be compared with early goal-directed therapy efforts to treat sepsis and septic shock [21]. The bundled interventions that significantly improved mortality in sepsis included individual elements, such as transfusion of red cells until hematocrit was >30%, that we no longer use today. This paper, so important to the early detection and management of sepsis, was at the core of the surviving sepsis campaign; however, once the bundled elements were studied individually, it was clear that not all the pieces of the bundle were beneficial. Analogously, the original ERAS protocol that bundled several perioperative interventions together to significantly improve surgical outcomes deserves to be broken down to identify which elements of the bundle positively impact outcomes and which do not. To that end, understanding the PCL effect on GV is paramount to its inclusion in the ERAS bundle and to amending traditional pre-surgical fasting guidelines.

In a large-scale prospective study, the administration of a PCL reduced hyperglycemia in non-diabetic surgical patients [22]. However, elevated GV, which is more harmful than hyperglycemia alone, has not been studied on such a large scale, nor in patients with type 2 DM. One of the reasons that GV has not been studied on a large scale is that there is no single consensus measurement for GV. This study measured GV in two different ways (CV and GLI); however, other GV measures have been studied, and there is no agreement on which measure best reflects GV. Even so, PCL ingestion appears to be safe and improves patient satisfaction when compared with traditional NPO guidelines, which may make it reasonable to continue to prescribe to surgical patients [22] even if a reduction in GV is not achieved.

Postoperative pain scores trended lower in the PCL group; however, they did not reach clinical significance. The inability to reach the desired sample size due to the high exclusion rate likely played a role in this. Because of the retrospective nature of the study, providers were not mandated to obtain BGC measurements. Thus, many patients were excluded for having fewer than two BGC values available.

Incentive spirometry scores postoperatively were also clinically insignificant. Performing incentive spirometry after surgery is intended to reduce postoperative pulmonary complications. It is part of routine postoperative care at our institution, although it has never been studied in relation to PCL. Since prior studies on hand grip strength and pulmonary function measured by peak expiratory flow rate showed significant improvement after PCL, and because incentive spirometry data were readily available, we decided to look at incentive spirometry as a secondary outcome. We hypothesized that incentive spirometry would improve in the PCL group based on the prior studies that showed improved muscle strength and pulmonary function. Unfortunately, our retrospective data set showed no significant improvement in incentive spirometry with PCL; however, this result could also be related to the relatively small sample size that was included in the final cohort.

The methodological approach utilized in this retrospective review was designed to obtain a historical dataset that would accurately capture an important perioperative change in clinical practice, allowing patients to ingest a carbohydrate-rich drink up to 2 h prior to surgery.

There are limitations to this retrospective study to discuss. With any retrospective study, tight control over the collection of variables was not possible. In this study, the variability in timing and frequency of BGC measurements was a clear limitation. We collected all BGC measurements from the immediate postoperative period through postoperative day 3; however, we did not analyze the differences in BGC collection times, nor were we able to match BGC collection timing between cohorts. If BGC measurements were to be collected at similar time points for each patient, a cleaner dataset could have been obtained. Similarly, a minimum of two BGC measurements were required for inclusion in the final cohort so that a standard deviation could be calculated and used to calculate CV; however, the standard deviation may be increasingly accurate with a larger number of BGC measurements.

The inability to obtain retrospective data on perioperative practices that could affect our primary and secondary outcomes is another limitation of this study. For example, data on intraoperative fluid administration, estimated blood loss, and insulin administration were unavailable retrospectively. All of these factors could contribute to GV, and controlling for them in a prospective study is paramount. Furthermore, the time difference of 3 years between the two cohorts of patients is a methodological limitation of this retrospective study. However, the time difference was unavoidable, as it was intended to provide a control and an intervention group corresponding to patients who underwent gynecological surgery before and after the institutional adoption of a new practice protocol. Finally, our sample size calculation suggested a sample size of 126 patients in each group to detect a 10% difference with 80% power; however, after exclusions, we did not reach that sample size. This may limit the generalizability of the findings to larger cohorts.

## 5. Conclusions

This retrospective review is one of the few studies to investigate the PCL effect on its intended outcome, a reduction in postoperative GV. This study reported GV in two ways, CV and GLI; however, other ways of calculating GV have been described. This study highlights the need for additional GV research, including consensus agreement on a gold standard GV measurement. Large-scale prospective studies are needed to test the effectiveness of PCL in reducing GV. Eventually, if risk factors for high GV can be determined and it is proven that PCL improves GV, patients with GV risk factors can be identified and prescribed a PCL prior to surgery.

## Figures and Tables

**Table 1:** Demographic characteristics and risk factors.

Data	Control (*n* = 45)	Intervention (*n* = 63)	*p*-Value
Age (years)	54.4	52.6	0.45
Race			0.59
White (%)	11 (24.4%)	13 (20.6%)	
African American (%)	22 (48.9%)	27 (42.9%)	
Hispanic or Latino (%)	6 (13.3%)	11 (17.5%)	
Asian (%)	2 (4.4%)	1 (1.6%)	
Not Available (%)	4 (8.9%)	11 (17.5%)	
Comorbidities			
Hypertension (%)	30 (66.7%)	32 (50.8%)	0.15
Diabetes Mellitus (%)	29 (64.4%)	29 (46.0%)	0.09
Body Mass Index (unit)	33.8	32.6	0.40
ASA Physical Status Classification			0.19
I	1 (2.2%)	0 (0%)	
II	23 (51.1%)	42 (67.7%)	
III	20 (44.4%)	19 (30.6%)	
IV	1 (2.2%)	1 (1.6%)	
Anesthetic Type			<0.001
TIVA	2 (4.4%)	5 (7.9%)	
Sevoflurane	36 (80.0%)	20 (31.7%)	
Propofol + Sevoflurane	7 (15.6%)	38 (60.3%)	

ASA = American Society of Anesthesiologists Physical Status, TIVA = total intravenous anesthetic.

**Table 2:** Results.

Variable	No PCL (*n* = 45)	PCL (*n* = 63)	*p*-Value
Mean BGC (mg/dL)	158.4	152.6	0.48
Standard Deviation	29.6	29.3	0.91
CV (%)	18.6	19.3	0.65
GLI	0.54	0.58	0.86
PACU Pain Score	5.2	4.5	0.23
PACU IS (mL)	1245	1262	0.87

BGC = blood glucose concentration, CV = coefficient of variance, GLI = glycemic lability index, PACU = postanesthesia care unit, IS = incentive spirometry.

**Table 3:** Subgroup analysis.

	No PCL (*n* = 45)	PCL (*n* = 63)	*p*-Value
DM Mean BGC (mg/dL)	168.5	171.5	0.77
DM CV (%)	17.8	19.3	0.49
DM GLI	0.65	1.08	0.34
Non-DM Mean BGC (mg/dL)	140.2	136.5	0.76
Non-DM CV (%)	20.0	19.4	0.85
Non-DM GLI	0.33	0.15	0.35

PCL = preoperative carbohydrate load, DM = diabetes mellitus, BGC = blood glucose concentration, CV = coefficient of variance, GLI = glycemic lability index.

## Data Availability

Data access is restricted due to privacy and ethical considerations.

## Appendix A. Perioperative ERAS Checklist: Gynecologic and Gyn-Oncologic Surgery

**ERAS:** PONV Screening Tool				
STEP 1	How many PONV risk factors present?			
**SCREENING**				
	**PONV Risk Factors**		**Points**	
	History of PONV		1	
	Motion sickness		1	
	Female gender		1	
	Non-smoker		1	
	Age <50 years		1	
	Use of opioids in PACU expected		1	
	**Total:**		**6**	
STEP 2	Use preop PONV risk factors to select PONV prophylaxis?			
**PONV RISK STRATIFICATION AND PROPHYLAXIS**				
**Total PONV Risk Factors**	**0–1**	**2**	**3**	**≥4**
**PONV Risk Level**	**Minimal**	**Low**	**Moderate**	**High**
**PONV prophylaxis**	**-----**	**Dexamethasone (4 mg)**	**Scopolamine** **(1.5 mg patch, preop)** **+** **Dexamethasone** **(4 mg)**	**Scopolamine** **(1.5 mg patch, preop)** **+** **Dexamethasone** **(4 mg)** **+** **Ondansetron (4 mg)**

## Appendix B. Surgery Types/CPT Codes Included in Dataset

total abdominal hysterectomy	58150, 58152, 58200, 58210, 58240
total vaginal hysterectomy	58260–58294
lap-assisted vaginal hysterectomy	58550–58554
total laparoscopic hysterectomy	58541–58544, 58570–58573, 58575
supracervical hysterectomy	58180
